# Simple presoaking modulates phytochemical recovery, antioxidant capacity, and macrophage responses of onion skin extracts

**DOI:** 10.1016/j.fochx.2026.104402

**Published:** 2026-09-02

**Authors:** Bekri Melka Abdo, Doyoung Song, Hae Ju Kang, Jeong Yeon Im, In-Guk Hwang, Ae Jin Choi, Suhyun Kwon, Sung-Hyen Lee

**Affiliations:** a Department of Food Sciences, National Institute of Crop and Food Science, Rural Development Administration, Wanju-gun, Jeollabuk-do 55365, Republic of Korea; b Wendo Genet Natural Product Research Laboratory, Ethiopian Institute of Agricultural Research, Ethiopia

**Keywords:** Onion skin, Presoaking, Quercetin derivatives, Antioxidant activity, RAW 264.7 macrophages, By-product valorization

## Abstract

Onion skin is a phenolic-rich by-product, but practical preprocessing strategies for directing phytochemical recovery remain insufficiently defined. This study evaluated whether brief presoaking in water, 5% vinegar, 1% citric acid, a vinegar–citric acid mixture, or 0.3% H₂O₂ for 4–12 min altered subsequent methanol-reference and hot-water extracts. Extraction yield, phenolic and flavonoid indices, quercetin markers, UHPLC–Orbitrap HRMS profiles, DPPH activity, and RAW 264.7 responses were assessed. Presoaking solution affected all major variables more consistently than duration. Across treatments, yields ranged from 5.78 to 10.67% for methanol and 6.87–10.53% for hot-water extracts, yet higher yield did not indicate greater phenolic density. Methanol preferentially recovered quercetin aglycone, whereas hot water produced a more polar profile. The 0.3% H₂O₂ condition showed favorable antioxidant performance without marked loss of metabolic viability. However, mild oxidative preprocessing requires further validation of peroxide removal, safety, and regulatory compliance before practical food application.

## Highlights

• Brief presoaking shifted recovery more strongly than duration within 4–12 min.
• Methanol and hot water yielded compositionally distinct onion-skin fractions.
• Extract yield alone did not predict phenolic density or antioxidant potency.
• Hot-water extraction supports a food-compatible route for by-product upgrading.
• Presoaking enables targeted valorization while defining safety and scale-up needs.

## 1 Introduction

Onion (*Allium cepa* L.) is one of the most widely consumed and processed vegetables worldwide, generating substantial residues during peeling, trimming, and fresh-cut preparation. The dry outer skin is commonly discarded, although recent studies have consistently shown that onion peel/skin is a concentrated source of phenolic compounds, flavonoids, and quercetin-related derivatives with strong antioxidant relevance (Gorrepati et al., 2024; Joković et al., 2024; Kumar et al., 2022; Stoica et al., 2023). This compositional feature supports the evaluation of onion skin not merely as a waste stream, but as a chemically valuable by-product for upcycled food ingredient development.

The valorization of onion skin requires processing strategies that are simple, scalable, and compatible with future food applications. Recent studies on onion skin waste valorization have emphasized extraction and preprocessing approaches that align with circular-economy and biorefinery principles while preserving phenolic-rich fractions (Ligarda-Samanez et al., 2025; Trigueros et al., 2024). Solvent selection is particularly important because extraction efficiency depends on both solvent properties and the physical or chemical condition of the plant matrix. Organic solvents such as methanol are useful as analytical reference systems for profiling less polar flavonol aglycones and related compounds, whereas water-based extraction is more relevant for future food-compatible ingredient development. Previous studies have also shown that solvent composition strongly influences the recovery of onion-skin flavonols, phenolics, and antioxidant compounds (Ciardi et al., 2021; Piechowiak et al., 2020; Prelac et al., 2023).

Despite growing interest in onion-skin valorization, the role of simple presoaking treatments before extraction remains insufficiently characterized. Presoaking is often treated as a washing step, although it may alter tissue hydration, matrix permeability, diffusion of soluble constituents, and the release or retention of phenolics. Water, vinegar, and citric acid are mild, inexpensive options with potential relevance to food processing. Low-concentration H₂O₂ was included here as an experimental oxidative condition, not as a food-ready treatment; any practical use would require validated residue removal, safety assessment, and regulatory compliance. Recent work on greener extraction systems for onion by-products further underscores the need to reduce dependence on conventional organic solvents while maintaining phytochemical recovery (Jaganmohanrao, 2025). Accordingly, presoaking was evaluated as a controllable preprocessing variable rather than merely a washing operation.

**Fig. 1:**
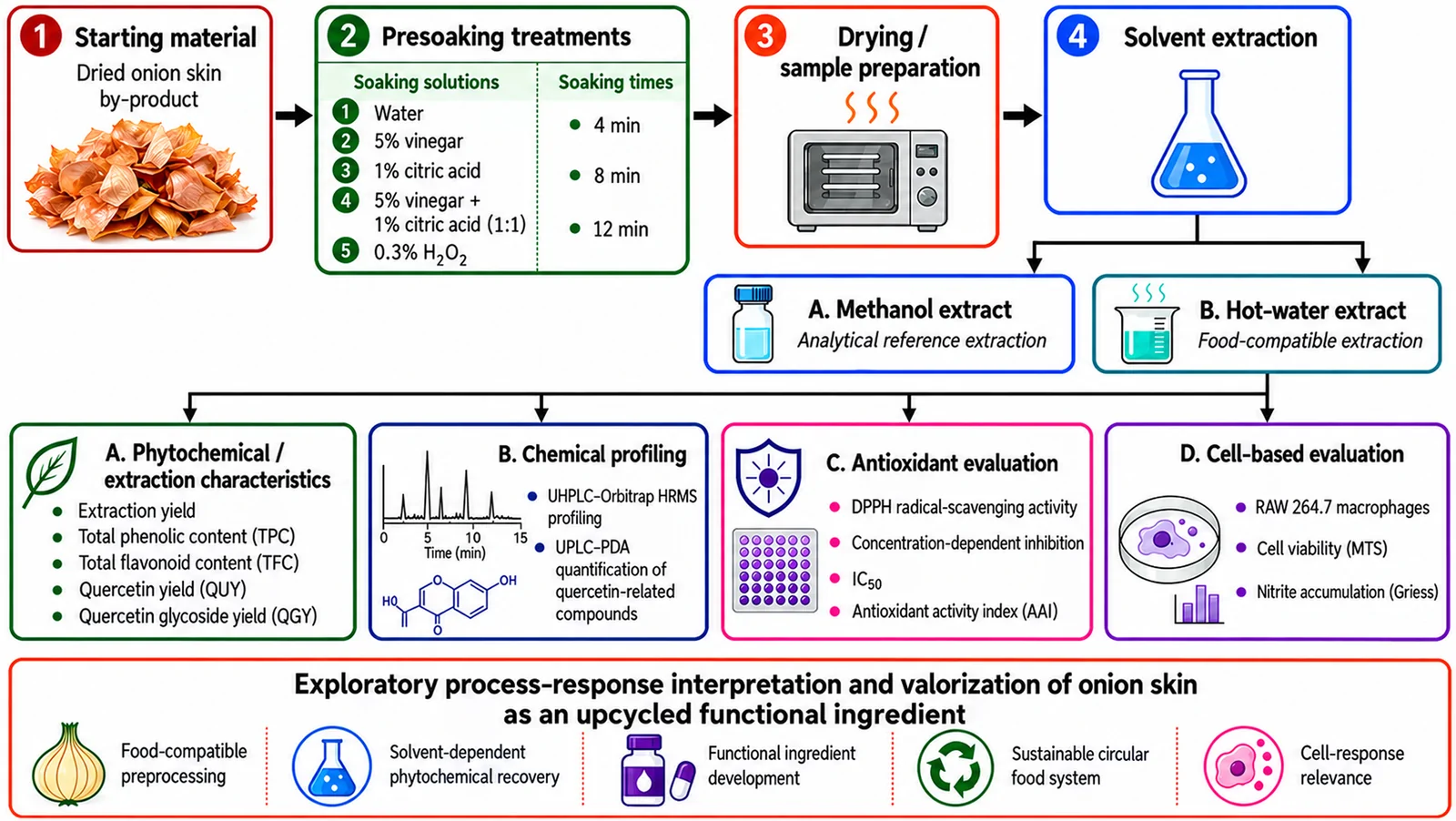
Experimental workflow for evaluating presoaking effects on phytochemical recovery, antioxidant activity, and macrophage responses of onion-skin extracts. Dried onion skins were presoaked in tap water, 5% vinegar, 1% citric acid, a 1:1 mixture of 5% vinegar and 1% citric acid, or 0.3% H₂O₂ for 4, 8, or 12 min. Water- and acid-based treatments were considered food-compatible, whereas H₂O₂ was evaluated as an experimental mild oxidative condition requiring further residue and safety validation. Samples were subsequently extracted with methanol as an analytical reference solvent or with hot water as a food-compatible extraction medium. Extraction yield, phytochemical indices, chromatographic profiles, antioxidant activity, and preliminary macrophage responses were evaluated.

This study therefore examined very short presoaking (4–12 min) as a distinct preprocessing step before two contrasting extraction systems. We tested whether presoaking solution would have a greater overall influence than duration within this narrow time range and whether its effects would depend on the subsequent extraction solvent. Extraction yield, phenolic and flavonoid indices, targeted quercetin markers, untargeted UHPLC–Orbitrap HRMS profiles, DPPH antioxidant potency, and preliminary RAW 264.7 macrophage responses were evaluated together. Methanol served as an analytical reference for recoverable flavonols, whereas hot water represented the more food-compatible route. The study was designed to support process selection according to extract composition and intended use, without implying industrial optimization, established safety, or therapeutic efficacy. The experimental workflow is summarized in Fig. 1.

## 2 Materials and methods

### 2.1 Onion skin and presoaking treatments

Dried onion skins (*Allium cepa* L.) were obtained from a regional producer and distribution organization in Mungyeong, Gyeongsangbuk-do, Republic of Korea. The material originated from conventionally cultivated yellow onions belonging to one medium-to-late-maturing hybrid cultivar lineage. The seed products were marketed domestically as ‘New Mars’ and ‘Katamaru’ and, according to supplier records, both derived from the imported cultivar ‘NEOEARTH’. The cultivar designation and country of origin were cross-checked against the official import declaration supplied by the seed importer, which identified NEOEARTH as a hybrid onion seed originating from the Netherlands. The 2024 crop was produced under conventional open-field conditions by contract farms in Yeongsun-myeon and Nongam-myeon, Mungyeong-si, and Pungyang-eup, Yecheon-gun, Gyeongsangbuk-do. Seeds were sown from late August to early September, seedlings were transplanted from October 20 to November 15, and bulbs were harvested from June 5 to June 25 of the following year. The experimental material consisted of relatively clean inner dry skins generated during commercial sorting and pooled from farms using the same cultivar lineage. Botanical identity was confirmed by the Mungyeong Agricultural Technology Center. Foreign material was removed manually, and intact skins were air-dried at 25 ± 2 °C to constant mass.

HPLC-grade methanol, acetonitrile, and formic acid were purchased from Merck (Darmstadt, Germany). Food-grade vinegar was obtained from Daesang Corporation (Seoul, Republic of Korea) and diluted to 5% (v/v) before use. Citric acid (Cat. No. 251275) and hydrogen peroxide solution (Cat. No. H1009) were purchased from Sigma-Aldrich (St. Louis, MO, USA) and diluted to 1% (w/v) and 0.3% (v/v), respectively. Ultrapure water was prepared using a Milli-Q purification system (Millipore, Bedford, MA, USA).

Onion skins were soaked at a material-to-solution ratio of 1:30 (w/v) in tap water, 5% vinegar, 1% citric acid, a 1:1 (v/v) mixture of 5% vinegar and 1% citric acid, or 0.3% hydrogen peroxide for 4, 8, or 12 min at 25 ± 2 °C. Water, vinegar, citric acid, and vinegar–citric acid were treated as food-compatible presoaking conditions. The 0.3% H₂O₂ condition was included as an experimental mild oxidative preprocessing strategy to determine whether controlled oxidation could modify matrix accessibility and phytochemical recovery. After presoaking, samples were rinsed with distilled water, air-dried to constant weight, and ground using a laboratory grinder (HMF-3260, Hanil Electric Co., Ltd., Republic of Korea). The resulting powder was stored in airtight containers at ambient temperature in an air-conditioned laboratory until extraction. Powdered onion skin was extracted at a solid-to-solvent ratio of 1:30 (w/v). Methanol extraction was performed by shaking at 310 rpm for 24 h at room temperature and served as an analytical reference for recoverable flavonols. Hot-water extraction was conducted at 95 °C for 2 h as the more food-compatible route. For the methanol-reference extraction, extracts were filtered through Whatman No. 1 filter paper and concentrated under reduced pressure using a rotary evaporator (EYELA N-1300, Tokyo Rikakikai Co., Japan) at ≤40 °C. For the hot-water extraction, samples were cooled to room temperature and centrifuged (Hanil Supra 22 K, Seoul, Republic of Korea) at 1500 rpm for 10 min at 4 °C, and the collected supernatants were freeze-dried using a PVTFD 10R freeze dryer (Ilsin Lab, Yangju, Republic of Korea). Extraction yield was calculated as dried-extract mass relative to the initial dry sample mass.

### 2.2 Phytochemical and chromatographic analyses

Total phenolic content was determined using the Folin–Ciocalteu method with minor laboratory-specific modifications (Singleton et al., 1999). Extract or gallic-acid standard solution (20 μL) was mixed with distilled water (80 μL) and Folin–Ciocalteu reagent (40 μL) in a 96-well plate. After 3 min, 60 μL of 10% (w/v) sodium carbonate was added, and the mixture was incubated for 1 h at room temperature in the dark. Absorbance was measured at 725 nm. Gallic acid standards (62.5–500 μg/mL) produced a calibration curve with R^2^ ≥ 0.99. Because this electron-transfer assay measures overall reducing capacity rather than phenolics exclusively, results were interpreted as a comparative total phenolic index and expressed as mg gallic acid equivalents (GAE)/g extract (Prior et al., 2005). Total flavonoid content was determined using the aluminum chloride colorimetric method with minor modifications (Pękal & Pyrzynska, 2014). Extract or quercetin standard solution (10 μL) was mixed with distilled water (40 μL), followed by 30 μL of 5% (w/v) sodium nitrite. After 5 min, 30 μL of 10% (w/v) aluminum chloride was added; after a further 6 min, 200 μL of 1 M sodium hydroxide was added. Absorbance was measured at 510 nm. Quercetin was used as the calibration standard over 62.5–1000 μg/mL (R^2^ ≥ 0.99), and results were expressed as mg quercetin equivalents (QE)/g extract. All measurements were performed in triplicate.

Major phenolic compounds were profiled using a Vanquish Flex UHPLC system coupled to an Orbitrap Exploris 120 high-resolution mass spectrometer (Thermo Fisher Scientific, Bremen, Germany). A 1 μL aliquot was injected, and mass spectrometric detection was conducted in negative electrospray ionization mode. Full-scan accurate-mass spectra were acquired over *m*/*z* 80–1200, and HCD MS/MS spectra were obtained with normalized collision energy set at 30%. Raw data were processed using Compound Discoverer version 3.4 (Thermo Fisher Scientific). Compound assignments were based on retention behavior, accurate mass, isotope pattern, characteristic fragment ions, authentic standards where available, and published onion-peel spectral data (Gorrepati et al., 2024; Joković et al., 2024). Compounds lacking direct confirmation with authentic standards were reported as tentatively identified. Quercetin and isoquercetin (quercetin-3-O-glucoside) were quantified by UPLC–PDA using a CORTECS C18 column (2.1 × 150 mm, 2.7 μm). A 2 μL aliquot of filtered extract solution (1 mg/mL) was injected. Mobile phases were 0.1% formic acid in water (A) and acetonitrile (B); the gradient was 10% B at 0 min, 25% B at 13 min, 50% B at 25 min, 90% B at 28–32 min, and 10% B at 35–45 min. The flow rate was 0.3 mL/min, the column temperature was 30 °C, and chromatograms were monitored at 350 nm. Peak identity was confirmed by retention-time comparison with authentic quercetin and isoquercetin standards. Each analyte was quantified using its corresponding external calibration curve (R^2^ > 0.99), and concentrations were expressed as mg/g extract.

### 2.3 Antioxidant activity assay

Antioxidant activity was evaluated using the DPPH radical-scavenging assay with minor laboratory-specific modifications (Brand-Williams et al., 1995). Extract solutions were prepared over the concentration range used for the concentration-response curves. A 10 μL aliquot of sample was mixed with 90 μL of freshly prepared 0.2 mM DPPH in methanol in a 96-well plate and incubated at 37 °C for 30 min in the dark. Absorbance was measured at 517 nm, with solvent blanks used for background correction and ascorbic acid included as a reference antioxidant. Radical-scavenging activity was expressed as percentage inhibition relative to the DPPH control. IC₅₀ values were estimated by four-parameter nonlinear regression, and the antioxidant activity index was calculated as the final DPPH concentration divided by the IC₅₀ value (Scherer & Godoy, 2009). Measurements were performed in triplicate. Because DPPH and Folin–Ciocalteu responses depend on assay chemistry and sample matrix, these endpoints were used for controlled treatment comparisons and were not interpreted as direct evidence of physiological antioxidant efficacy (Prior et al., 2005; Rumpf et al., 2023).

### 2.4 Macrophage-based screening assays

RAW 264.7 macrophages obtained from the Korean Cell Line Bank (KCLB, Seoul, Republic of Korea) were maintained in Dulbecco's modified Eagle's medium supplemented with 10% heat-inactivated fetal bovine serum and 1% penicillin–streptomycin at 37 °C in a humidified atmosphere containing 5% CO₂. Cells were allowed to attach for 24 h and were then exposed to extracts at 125, 250, or 500 μg/mL for a further 24 h. Metabolic viability was assessed by adding 20 μL of CellTiter 96® AQueous One Solution reagent (MTS; Promega, Madison, WI, USA), incubating for 4 h at 37 °C, and measuring absorbance at 490 nm. MTS reduction was interpreted as a metabolic cytocompatibility indicator rather than a direct measure of proliferation (Stockert et al., 2018). For nitrite determination, 100 μL of culture supernatant was mixed with 100 μL of Griess reagent, incubated for 10 min, and measured at 540 nm. Sodium nitrite was used for calibration, and the result was interpreted as an indirect index of nitric oxide formation (Green et al., 1982). Extracts were tested without co-treatment with an inflammatory stimulant; lipopolysaccharide was included only as a separate reference control. Experiments were conducted in three independent runs with technical triplicates. Accordingly, these assays were treated as preliminary macrophage-response screening rather than as evidence of a defined beneficial immune or inflammatory effect.

### 2.5 Exploratory response and correlation analyses

Exploratory contour analysis was used to visualize the combined influence of presoaking-solution pH and duration on DPPH IC₅₀ values. Because the solutions differed in chemical composition as well as pH, the plots were treated as descriptive process–response visualizations within the tested range and not as definitive pH optimization. Pearson correlation analysis was performed separately for methanol-reference and hot-water extracts using treatment-level observations to examine associations among extraction yield, phytochemical indices, quercetin-related markers, IC₅₀ values, and preliminary macrophage responses. Because the correlation matrices were based on a limited number of treatment-level observations, they were interpreted as exploratory and hypothesis-generating rather than as evidence of causality or mechanism.

For macrophage-based screening, one duration-concentration combination was selected for each extract type for concise visualization in the main figure. Selection was based on a measurable response range, replicate consistency, and avoidance of pronounced floor or ceiling effects within each solvent-specific dataset. Because the conditions were selected from exploratory screening data and differed between extract types, the resulting comparisons were restricted to presoaking treatments within each extract type and were not used for direct cross-solvent potency claims.

### 2.6 Statistical analysis

Data are presented as mean ± SD. Each presoaking-solution × duration combination was analyzed in triplicate (*n* = 3). The effects of presoaking solution and duration were evaluated by two-way ANOVA. When an overall effect was significant, Fisher's least significant difference (LSD) test was used for treatment-wise comparisons. IC₅₀, AAI, and macrophage-screening outcomes were analyzed within each extract type using one-way ANOVA followed by the LSD test. Because significant solution × duration interactions occurred for several phytochemical variables, main-effect means were interpreted together with the interaction results in Table S1. Analyses were performed using R version 4.3.1 (R Foundation for Statistical Computing, Vienna, Austria), GraphPad Prism version 9.0 (GraphPad Software, San Diego, CA, USA), and Minitab version 19 (Minitab LLC, State College, PA, USA). Differences were considered significant at *p* < 0.05.

## 3 Results and discussion

### 3.1 Effects of presoaking treatments on extraction yield and phytochemical recovery

Presoaking produced clear solution- and solvent-dependent differences in extraction yield and phytochemical recovery (Table 1). Two-way ANOVA confirmed a significant main effect of soaking solution for every major parameter, whereas soaking duration affected fewer variables and several solution × duration interactions remained significant (Table S1). Thus, within the short 4–12 min range, solution chemistry was generally a stronger determinant than duration alone, although the significant interactions indicate that a single universal time effect should not be assumed. This pattern is consistent with studies showing that onion-skin phenolic recovery is strongly governed by solvent polarity, temperature, and matrix pretreatment (Ciardi et al., 2021; Piechowiak et al., 2020; Prelac et al., 2023; Trigueros et al., 2024).

**Table 1:** Effects of different presoaking treatments and durations on extraction yield and phytochemical composition of onion skin extracts.

A. Soaking solution (pH)	EXY (%)		TPC (mg GAE/g)		TFC (mg QE/g)		QUY (mg/g)	QGY (mg/g)	
	Methanol	H. water	Methanol	H. water	Methanol	H. water	Methanol	Methanol	H. water
Water (6.48)	5.78 ± 0.60^d^	6.95 ± 1.35^c^	490.8 ± 56.6^a^	163.3 ± 12.6^a^	794.5 ± 102.5^ab^	864.1 ± 64.9^b^	135.0 ± 13.4^a^	49.9 ± 2.7^e^	32.0 ± 0.5^d^
5% vinegar (2.72)	6.56 ± 0.62^c^	7.28 ± 0.67^c^	478.6 ± 45.6^ab^	161.4 ± 14.8^a^	755.2 ± 110.4^b^	855.9 ± 61.9^b^	145.0 ± 11.3^a^	65.6 ± 3.7^c^	37.6 ± 0.7^a^
1% Citric acid (2.12)	10.67 ± 0.87^a^	10.53 ± 1.23^a^	398.7 ± 23.0^d^	82.6 ± 12.5^c^	821.5 ± 146.2^a^	213.6 ± 71.3^d^	95.2 ± 6.1^c^	56.9 ± 2.9^d^	31.3 ± 1.1^e^
5% vinegar & 1% citric acid (2.42)	9.44 ± 0.82^b^	9.62 ± 0.90^b^	414.1 ± 7.5^c^	101.9 ± 15.0^b^	645.4 ± 164.6^c^	323.0 ± 58.8^c^	108.8 ± 7.9^b^	74.2 ± 3.8^a^	36.6 ± 1.5^c^
0.3% hydrogen peroxide (5.76)	6.33 ± 0.29^cd^	6.87 ± 0.50^c^	466.7 ± 64.5^b^	163.6 ± 13.1^a^	575.3 ± 74.5^d^	918.4 ± 75.4^a^	140.1 ± 12.3^a^	70.9 ± 1.6^b^	38.5 ± 0.9^a^
LSD (α = 0.05)	0.5564	0.8904	13.684	9.17	55.184	48.285	9.979	1.197	0.119

B. Soaking time (min)	EXY (%)		TPC (mg GAE/g)		TFC (mg QE/g)		QUY (mg/g)	QGY (mg/g)	
	Methanol	H. water	Methanol	H. water	Methanol	H. water	Methanol	Methanol	H. water
4	7.80 ± 2.06^NS^	8.29 ± 2.11^NS^	486.2 ± 69.2^a^	133.8 ± 37.2^NS^	693.4 ± 145.3^b^	647.1 ± 320.0^NS^	127.8 ± 22.9^NS^	63.8 ± 10.7^a^	35.3 ± 3.7^b^
8	7.87 ± 2.44	8.26 ± 1.57	415.4 ± 11.9^c^	135.2 ± 37.7	751.8 ± 192.7^a^	637.6 ± 303.0	123.6 ± 21.7	62.6 ± 8.0^b^	35.5 ± 3.4^a^
12	7.60 ± 1.75	8.20 ± 1.82	447.7 ± 49.1^b^	134.6 ± 41.1	709.9 ± 108.1^ab^	620.3 ± 334.1	123.0 ± 22.7	64.1 ± 10.2^a^	34.8 ± 2.4^c^
LSD (α = 0.05)	0.431	0.6897	10.6	7.103	42.746	37.401	7.73	0.927	0.092

For soaking solutions, values are presented as mean ± SD (*n* = 9). For soaking duration, values are presented as marginal means ± SD across the five soaking solutions at each soaking time (*n* = 15). EXY, extraction yield (%); TPC, total phenolic content (mg GAE/g extract); TFC, total flavonoid content (mg QE/g extract); QUY, quercetin yield (mg/g extract); QGY, quercetin glycoside yield (mg/g extract); H. water, hot-water extract. Different letters within a column indicate significant differences at α = 0.05. NS, not significant.

Citric acid and the vinegar–citric acid mixture produced the highest extraction yields in both methanol and hot-water extracts. Acidic hydration and matrix softening may have increased the release of soluble solids, but the present experiment did not directly measure structural changes in the skin matrix. The greater mass yield was not accompanied by the highest phenolic index, suggesting that additional non-phenolic constituents were also recovered. Similar divergence between total-solids recovery and phenolic density has been reported for onion-peel extraction with ethanol, hot water, and subcritical water (Benito-Román et al., 2020; Lee et al., 2014; Trigueros et al., 2024). Extraction yield alone is therefore insufficient for process selection; compositional markers and functional performance should be considered together. Although the pooled commercial lot was traceable to one cultivar lineage, environmental effects cannot be excluded, as genotype × environment interactions have been reported for Korean-grown onion cultivars including Katamaru (Imran et al., 2025).

The phytochemical responses differed clearly between extraction solvents. Methanol generally favored phenolic- and flavonol-rich fractions, whereas hot-water extraction was more sensitive to presoaking chemistry. Among the tested conditions, 0.3% H₂O₂ was associated with high hot-water TFC and favorable DPPH potency, which is consistent with a change in matrix accessibility or release of antioxidant-active constituents. Because matrix disruption and oxidative transformation products were not measured, the mechanism cannot be assigned from the present data. The H₂O₂-presoaked extracts did not markedly reduce RAW 264.7 metabolic viability under the screening conditions, but this observation does not establish food-use safety. Residual peroxide, removal efficiency, oxidation products, and regulatory compliance must be evaluated before the process can be considered for food application. Studies of mild oxidative pretreatment in lignocellulosic matrices provide a useful mechanistic analogy for enhanced accessibility, although they do not directly validate the present onion-skin process (Brienza et al., 2024).

### 3.2 Solvent-dependent phytochemical profiles

The chromatographic data indicate that methanol and hot water generated compositionally distinct fractions rather than different quantities of an identical extract (Table 2; Fig. S1). Because electrospray ionization efficiency differs among compounds, TIC-normalized percentages were interpreted as within-extract signal distributions and not as molar composition or absolute concentration. Methanol was used to characterize the analytically recoverable flavonol reservoir, whereas hot water represented the more food-compatible route and yielded a broader polar profile. This solvent-selective fractionation agrees with onion-skin studies in which methanol or aqueous alcohol preferentially recovered quercetin-rich fractions, whereas hot or subcritical water increased the recovery of more polar phenolics and organic acids (Ciardi et al., 2021; Joković et al., 2024; Prelac et al., 2023; Trigueros et al., 2024). The importance of distinguishing crude recovery from selective enrichment is also supported by resin-based processing of red onion peel, in which optimized adsorption increased the concentration of major polyphenols and enhanced antioxidant responses relative to the crude extract (Aliya et al., 2025). Although downstream purification differs from presoaking, both approaches show that compositional quality, not extract mass alone, should guide valorization decisions.

**Table 2:** Major constituents of onion-skin extracts tentatively identified by UHPLC–Orbitrap HRMS analysis (ESI− mode).

No.	Compound	Molecular formula	RT (min)	Relative abundance (%)†	
				Methanol extract	Hot water extract
1	Hex-2-ulose	C_6_H_12_O_6_	0.887	1.57	2.91
2	Galacturonic acid	C_6_H_10_O_7_	0.912	0.23	2.03
3	DL-Lactic Acid	C_3_H_6_O_3_	1.366	1.65	2.21
4	Citric acid	C_6_H_8_O_7_	1.504	1.82	26.67
5	Protocatechuic acid	C_7_H_6_O_4_	4.538	5.90	35.77
6	Catechol	C_6_H_6_O_2_	4.540	0.46	2.64
7	Isoquercetin	C_21_H_20_O_12_	6.829	16.35	13.52
8	Quercetin	C_15_H_10_O_7_	7.710	61.10	4.95
9	Luteolin	C_15_H_10_O_6_	8.359	3.89	0.24
10	Isorhamnetin	C_16_H_12_O_7_	8.467	3.44	0.33
11	(−)-Pinellic acid	C_18_H_34_O_5_	9.998	2.18	3.49

† Relative abundance (%) was calculated by peak-area normalization within each extract based on total ion chromatogram intensity and represents a semi-quantitative within-extract signal distribution rather than absolute concentration. Compounds were assigned using accurate mass, isotope pattern, retention behavior, HCD MS/MS fragments, authentic standards where available, and published spectral evidence. Assignments lacking confirmation with authentic standards are reported as tentative. Both extract profiles were obtained from onion skins presoaked in 5% vinegar for 8 min.

Quercetin and isoquercetin were selected as targeted flavonol markers because quercetin and its glycosides are repeatedly identified as dominant flavonols in onion skins (Gorrepati et al., 2024; Joković et al., 2024; Stoica et al., 2023). UPLC–PDA analysis showed that quercetin aglycone was recovered predominantly in methanol extracts, consistent with its limited aqueous solubility and greater affinity for organic-solvent systems. By contrast, hot-water extracts showed relatively stronger protocatechuic- and citric-acid signals. The Orbitrap HRMS signal distributions were semi-quantitative within each extract and were not equated with absolute compound concentrations. The compositional differences also reinforce the importance of cultivar disclosure because onion-skin genotype and growing environment can substantially affect flavonoid abundance and antioxidant responses (Gorrepati et al., 2024; Imran et al., 2025; Sagar et al., 2021). Accordingly, the value of the hot-water fraction should be judged by its overall polar composition, antioxidant performance, and suitability for the intended food matrix rather than by quercetin aglycone alone.

Taken together, the chromatographic results show that hot-water extraction should not be evaluated solely by quercetin aglycone recovery. Its broader polar profile, antioxidant response, and compatibility with future food-processing studies are equally relevant.

### 3.3 Effects of presoaking solutions on antioxidant activity

**Fig. 2:**
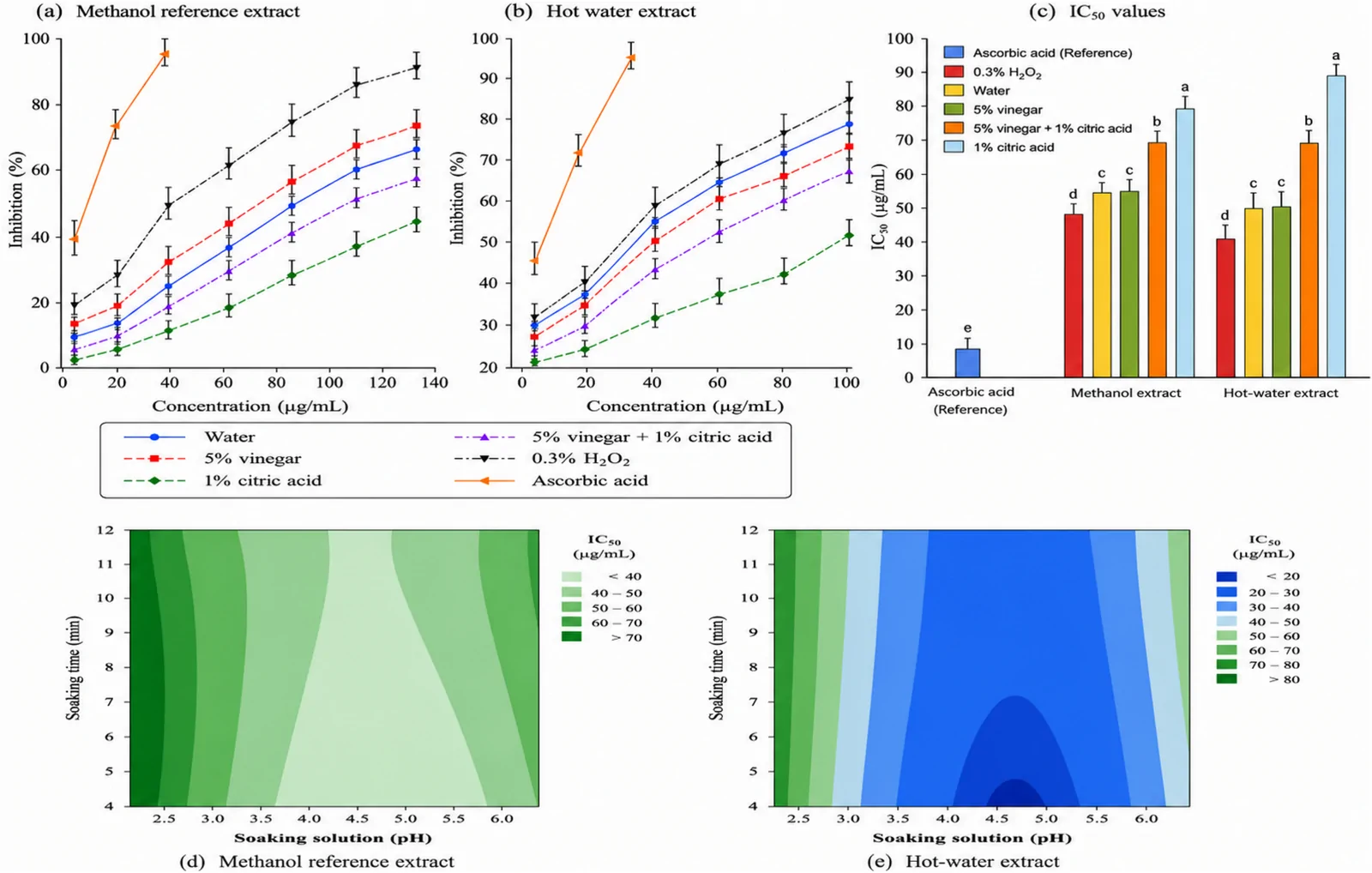
Antioxidant activity of onion skin extracts prepared using different presoaking treatments. DPPH radical-scavenging activity of (a) methanol reference extracts and (b) hot-water extracts. Extracts were tested across the final assay concentrations shown in the figure after mixing with DPPH solution, and radical-scavenging activity was expressed as percentage inhibition relative to the DPPH control without extract. (c) IC₅₀ values of DPPH radical-scavenging activity for methanol reference and hot-water extracts. Lower IC₅₀ values indicate stronger radical-scavenging potency. (d, e) Exploratory contour plots showing descriptive process–response patterns between presoaking solution pH, presoaking duration, and IC₅₀ values in methanol reference and hot-water extracts, respectively. For panels (a–c), values are presented as mean ± SD across the three presoaking durations (4, 8, and 12 min), with each duration analyzed in triplicate (n = 9). In panel (c), ascorbic acid was included as a reference comparator, and different letters indicate significant differences among the compared IC₅₀ groups (*p* < 0.05).

The antioxidant potency of onion-skin extracts varied with presoaking treatment and extraction solvent (Fig. 2). DPPH radical-scavenging activity increased with extract concentration in both methanol-reference and hot-water extracts (Fig. 2a and b), and IC₅₀ values differentiated treatment performance (Fig. 2c). The 0.3% H₂O₂ condition showed one of the most favorable antioxidant profiles. Mild oxidation may have increased access to antioxidant-active constituents through matrix modification or release, but the present data do not distinguish enhanced extraction from chemical conversion or degradation. Onion-skin flavonols, particularly quercetin and its derivatives, have well-established radical-stabilizing properties (Fredotović et al., 2021; Joković et al., 2024; Prelac et al., 2023), while cultivar-dependent composition can substantially influence assay outcomes (Gorrepati et al., 2024). DPPH results should therefore be interpreted alongside compositional and cell-based measurements rather than as direct physiological efficacy (Prior et al., 2005).

Extraction yield did not consistently predict antioxidant potency, indicating that extract composition was more informative than recovered mass alone. The contour plots showed combined process–response patterns involving solution chemistry, measured pH, and soaking duration within the tested range (Fig. 2d and e). Because the presoaking solutions differed chemically as well as in pH, the plots were interpreted descriptively and were not used to claim a general pH optimum.

### 3.4 Solvent-specific RAW 264.7 macrophage responses

**Fig. 3:**
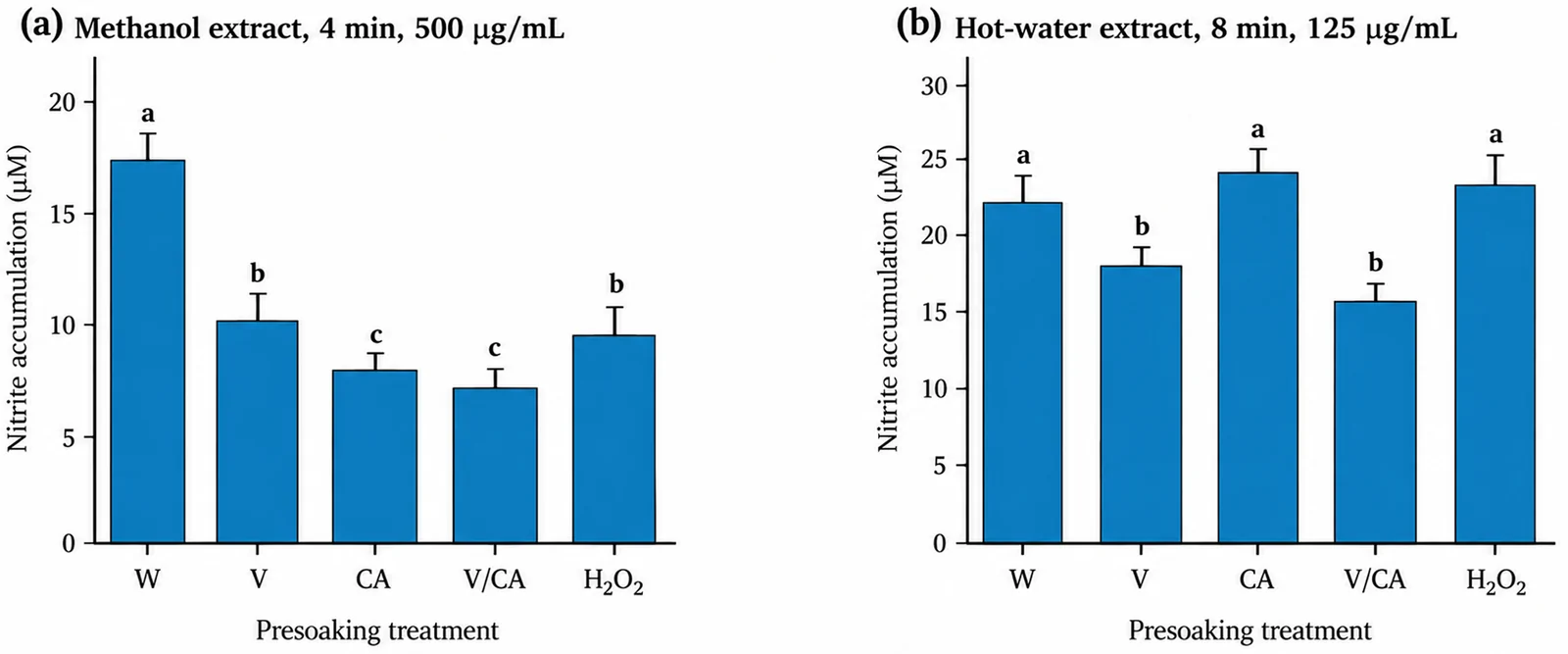
Nitrite accumulation in RAW 264.7 macrophage cultures treated with onion-skin extracts prepared under representative presoaking conditions. (a) Nitrite accumulation after treatment with methanol extracts prepared following 4 min of presoaking and tested at 500 μg/mL. (b) Nitrite accumulation after treatment with hot-water extracts prepared following 8 min of presoaking and tested at 125 μg/mL. W, water; V, vinegar; CA, citric acid; V/CA, vinegar–citric acid mixture; H₂O₂, hydrogen peroxide. Values are mean ± SD. Different letters indicate significant differences among presoaking treatments within each extract type (*p* < 0.05). Nitrite was measured by the Griess reaction as an indirect index of nitric oxide production.

Selected onion-skin extracts were screened in RAW 264.7 macrophages as a preliminary cell-response endpoint (Fig. 3). The extracts did not markedly reduce MTS metabolic activity under the tested conditions, supporting their use in subsequent nitrite screening. However, MTS reduction reflects cellular metabolic activity and is not a direct measure of proliferation, absence of cytotoxicity by all mechanisms, or comprehensive safety (Stockert et al., 2018). Nitrite accumulation measured by the Griess reaction is an indirect index of nitric oxide formation (Green et al., 1982), and its biological meaning depends on dose, exposure conditions, activation state, and signaling context.

The representative conditions shown in Fig. 3 were 4 min and 500 μg/mL for methanol extracts and 8 min and 125 μg/mL for hot-water extracts (Fig. S2). These solvent-specific conditions were chosen to display treatment separation within a measurable response range. They permit comparison among presoaking treatments within an extract type but do not support direct potency comparison between methanol and hot-water extracts.

Presoaking-dependent differences in nitrite accumulation indicate that extract composition affected macrophage responses under the selected conditions. However, the direction of a nitrite response cannot be classified as beneficial without a defined inflammatory model. The extract types were tested at different representative doses and durations, and no mechanistic challenge model was used. Although quercetin can suppress lipopolysaccharide-induced nitric oxide production in activated RAW 264.7 cells (Mu et al., 2001), that experimental context differs from the present screening design. These results should therefore guide, rather than replace, subsequent studies using standardized exposure conditions, appropriate controls, inflammatory challenges, cytokine measurements, and pathway-level endpoints. Recent work on hydroethanolic onion-skin extracts combined compositional profiling with cell-viability and inflammation-related cellular assays, including activated BV2 microglia, illustrating how biological interpretation depends on a defined challenge model and matched exposure conditions (Trigueros et al., 2025).

### 3.5 Implications for onion-skin valorization

The main practical implication is that presoaking should be treated as a process variable rather than as an inconsequential washing step. Even within 4–12 min, the soaking solution changed extract composition and functional readouts, and the preferred condition depended on the extraction solvent and target profile. A short ambient-temperature pretreatment followed by hot-water extraction may be compatible with existing food-processing operations and could support the use of onion skin as a standardized ingredient source. These potential benefits, however, have not yet been demonstrated at production scale. Pilot studies should quantify material yield, water and energy use, residue management, lot-to-lot consistency, product quality, and processing cost before environmental or economic advantages are claimed. Process selection should therefore consider chemical markers and functional performance together with safety, solvent use, energy demand, intended product, and downstream requirements (Bains et al., 2023; Jaganmohanrao, 2025; Kyei et al., 2025; Ligarda-Samanez et al., 2025; Stoica et al., 2023; Trigueros et al., 2024).

The exploratory correlation matrices showed solvent-specific associations among phytochemical indices, DPPH potency, and representative nitrite responses (Fig. S3). Because these analyses used a limited number of treatment-level means, they should be interpreted as descriptive and hypothesis-generating rather than confirmatory. The contrasting patterns further reinforce that methanol and hot water recovered different chemical fractions and that biological responses cannot be attributed to a single marker without targeted validation. Methanol served as an analytical benchmark, whereas hot water is the more relevant route for future food-ingredient development. A practical development pathway would include selection of a target compositional profile, confirmation across harvests and commercial lots, pilot-scale extraction and mass-balance studies, stability and sensory evaluation, food-matrix performance, safety testing, regulatory assessment, and techno-economic analysis. For any H₂O₂-assisted process, peroxide-removal efficiency, residual peroxide, oxidation products, batch-to-batch composition, and worker and environmental controls must also be established. Recent bread studies illustrate why extract activity alone cannot predict technological behavior in a formulated food (Wang et al., 2025). Thus, the present work provides a process-selection framework and testable scale-up priorities rather than evidence of industrial readiness.

## 4 Conclusions

Brief presoaking altered onion-skin extraction in a treatment- and solvent-dependent manner. Solution chemistry exerted a stronger overall influence than duration within the 4–12 min range, and extraction yield did not consistently reflect phenolic density or antioxidant potency. Methanol preferentially recovered a flavonol-rich fraction, whereas hot water yielded a compositionally distinct, more polar extract, showing that preprocessing and solvent choice can be used to direct recovery toward different chemical profiles. The 0.3% H₂O₂ condition showed favorable antioxidant performance without a marked reduction in RAW 264.7 metabolic viability under the selected screening conditions, but it remains an experimental oxidative treatment rather than a food-ready process. The results support further development of hot-water onion-skin extracts with defined compositional targets. Translation will require confirmation across raw-material lots, pilot-scale mass and energy balances, control of peroxide and oxidation products where relevant, compositional specifications, safety and stability testing, sensory and food-matrix evaluation, regulatory review, and techno-economic assessment.

## Declaration of generative AI use

During the preparation of this work, the authors used OpenAI ChatGPT to improve language, readability, manuscript organization, and internal consistency. After using this tool, the authors reviewed and edited the content as needed, verified the experimental descriptions and conclusions, and take full responsibility for the content of the article.

## CRediT authorship contribution statement

**Bekri Melka Abdo:** Writing – original draft, Methodology, Formal analysis. **Doyoung Song:** Writing – review & editing, Writing – original draft, Methodology. **Hae Ju Kang:** Methodology, Formal analysis, Validation, Writing – review & editing. **Jeong Yeon Im:** Software, Methodology, Formal analysis, Validation. **In-Guk Hwang:** Validation, Methodology. **Ae Jin Choi:** Resources, Investigation. **Suhyun Kwon:** Methodology, Formal analysis. **Sung-Hyen Lee:** Writing – review & editing, Writing – original draft, Visualization, Validation, Supervision, Resources, Project administration, Methodology, Investigation, Funding acquisition, Data curation, Conceptualization.

## Funding

This research was funded by 10.13039/501100003627Rural Development Administration, Republic of Korea (grant number PJ01770401).

## Declaration of competing interest

The authors declare that they have no known competing financial interests or personal relationships that could have appeared to influence the work reported in this paper.

## Data Availability

Data will be made available on request.
