# Supplementary material for: Simple presoaking modulates phytochemical recovery, antioxidant capacity, and macrophage responses of onion skin extracts
Source: Food Chem X. 2026 Sep 2;39:104402. doi: 10.1016/j.fochx.2026.104402 (PMC13579916; doi:10.1016/j.fochx.2026.104402)
Supplement: Supplementary file 1 [file mmc1.docx]

|  |  |
| --- | --- |

**Fig. S1. Representative chromatographic profiles of onion skin extracts prepared using methanol and hot water**

A. UHPLC–Orbitrap HRMS base peak chromatograms acquired in negative electrospray ionization mode. Peak numbers correspond to the tentatively identified compounds listed in Table 2. B. UPLC–PDA chromatograms monitored at 350 nm. Major peaks were assigned as quercetin-3-O-glucoside (isoquercetin) and quercetin by retention-time comparison with authentic standards; quantification used the corresponding external calibration curves.

**Fig. S2. Selection of representative conditions for macrophage-based nitrite screening.**

Mean nitrite accumulation across presoaking treatments is summarized by presoaking duration and extract concentration. Conditions for Fig. 3 were selected to provide a measurable response range with acceptable replicate consistency within each extract type. The selected conditions were 4 min at 500 μg/mL for methanol extracts and 8 min at 125 μg/mL for hot-water extracts. These exploratory selections were used for within-extract visualization and not for direct cross-solvent potency comparison.

**Fig. S3. Pearson correlations among phytochemical variables, antioxidant activity, and nitrite accumulation in onion-skin extracts prepared with methanol or hot water**

(a) Pearson correlation matrix for methanol-reference extracts. (b) Pearson correlation matrix for hot-water extracts. Variables included extraction yield, total phenolic index, total flavonoid content, quercetin-related compound yields, DPPH IC_50_, and nitrite accumulation. EXY, extraction yield; TPC, total phenolic index; TFC, total flavonoid content; QUY, quercetin yield; QGY, quercetin glycoside yield; IC_50_, half-maximal inhibitory concentration in the DPPH assay. QUY was included for methanol extracts, whereas the hot-water analysis used QGY because quercetin was below the quantification limit under the present UPLC-PDA conditions. Nitrite values were treatment-level means obtained under the representative conditions shown in Fig. 3: 4 min at 500 μg/mL for methanol extracts and 8 min at 125 μg/mL for hot-water extracts. Correlations were exploratory and were not interpreted as causal relationships.
